# Fine Particulate Matter in Urban Environments: A Trigger of Respiratory Symptoms in Sensitive Children

**DOI:** 10.3390/ijerph13121246

**Published:** 2016-12-15

**Authors:** Daniel Dunea, Stefania Iordache, Alin Pohoata

**Affiliations:** 1 Faculty of Environmental Engineering and Food Science, Valahia University of Targoviste, Aleea Sinaia No.13, RO-130004 Targoviste, jud. Dambovita, Romania; 2 Faculty of Sciences and Arts, Valahia University of Targoviste, Bd. Unirii No.18-24, RO-130082 Targoviste, jud. Dambovita, Romania; alinpohoata@yahoo.com

**Keywords:** PM_2.5_, multi-criteria approach, estimated dose, respiratory health, wheezing, fever, outdoor air quality

## Abstract

The overall objective of this research was to study children’s respiratory illness levels in Targoviste (Romania) in relationship to the outdoor concentrations of airborne particulate matter with an aerodynamic diameter below 2.5 µm (PM_2.5_). We monitored and analysed the PM_2.5_ concentrations according to a complex experimental protocol. The health trial was conducted over three months (October–December 2015) and required the active cooperation of the children’s parents to monitor carefully the respiratory symptoms of the child, i.e., coughing, rhinorrhoea, wheezing, and fever, as well as their outdoor program. We selected the most sensitive children (*n* = 25; age: 2–10 years) with perturbed respiratory health, i.e., wheezing, asthma, and associated symptoms. The estimated average PM_2.5_ doses were 0.8–14.5 µg·day^−1^ for weekdays, and 0.4–6.6 µg·day^−1^ for the weekend. The frequency and duration of the symptoms decreased with increasing age. The 4- to 5-year old children recorded the longest duration of symptoms, except for rhinorrhoea, which suggested that this age interval is the most vulnerable to exogenous trigger agents (*p* < 0.01) compared to the other age groups. PM_2.5_ air pollution was found to have a direct positive correlation with the number of wheezing episodes (*r* = 0.87; *p* < 0.01) in November 2015. Monitoring of wheezing occurrences in the absence of fever can provide a reliable assessment of the air pollution effect on the exacerbation of asthma and respiratory disorders in sensitive children.

## 1. Introduction

Recent studies on urban air pollution showed that long-term exposure to high levels of contaminants is a direct cause of major adverse health effects in air-polluted areas [1,2]. According to World Bank estimates, approximately 800,000 people die prematurely every year from lung cancer or cardiovascular and respiratory diseases caused by outdoor air pollution. The safe levels of exposure to the combined presence of air contaminants below which no adverse health effects occur are difficult to establish [3]. Children are particularly vulnerable to atmospheric pollution because of their elevated metabolic rates, physiological and anatomical immaturity, and intense daily outdoor activity [4]. Recent studies showed that the lung function of children resulted in acute but reversible decrease after pollution episodes of ozone and particulate matter (PM) [5,6]. Children’s vulnerability to air pollution is high because their respiratory system is not completely developed, consisting of sensitive lung tissue [7,8]. Furthermore, children often use mouth breathing, which substantially reduces the filtering provided by the nasal cavity compared with nose breathing [9].

Most frequent respiratory effects in children, which likely have a causal relationship, are considered to result in respiratory-related hospital admissions and asthma-related visits to the emergency department [10]. Acute respiratory infections are a major cause of morbidity and mortality among children [11,12]. Although respiratory infections are common in children, a specific diagnosis is rare. Consequently, most epidemiological studies that have established a link with air pollution are based on respiratory symptoms [13]. The most frequent clinical diagnostics recorded from hospital admissions are bronchiolitis, interstitial acute pneumonia with wheezing, recurrent wheezing and asthma [14]. Wheezing is a key respiratory symptom for assessing asthma attacks due to exogenous triggers. When intra-thoracic airways are obstructed, positive pleural pressure becomes higher in expiration, and airways collapse in expiration occurs downstream of the obstruction, producing an exaggeration of the obstruction with an audible expiratory whistling (wheezing) and a limitation of expiratory flow [15]. Asthma is a chronic disease with unpredictable evolution and can cause considerable restrictions that have emotional and social impacts on the life of the whole family. Children with asthma present with limitations in physical activity and school absenteeism. The consequence of these issues, along with increased asthma incidence, and the severity of clinical forms, is seen in the rising cost of care and treatment for these children [16], impacting negatively on society.

Implementing measures to prevent and control asthma and other chronic diseases in childhood should be a priority for the medical and social factors in each country. The need to control asthma in children requires access to comprehensive and reliable information on pollution in the specific microenvironments where children live. Moreover, a long-term cooperation among authorities, air quality specialists and paediatricians is required to improve the knowledge of air pollution effects on children’s health by monitoring the respiratory diseases classified by the International Classification of Diseases (ICD-10-WHO 2016), which are potentially caused or aggravated by atmospheric pollution [14].

Recent evidence has shown that fine (PM_2.5_—particulate matter with an aerodynamic diameter below 2.5 µm) and ultrafine (PM_0.1_—diameter below 0.1 µm) fractions of airborne PM, which contain many harmful components such as black smoke, PAHs, metals and inorganic salts, are mostly responsible for adverse health effects in urban environments [1]. There is a clear need for further research to establish a link between the PM levels/associated chemical speciation and medical evidence to support conclusions, because many approaches have used only statistical or empirical methods [17]. Studies of the quantitative health risk at high PM concentrations require the selection and delineation of urban study areas based on several criteria as follows: the availability of sufficient air quality data, the inclusion in an epidemiology study, and the availability of baseline incidence data regarding the health effects in a specific microenvironment [18]. It was demonstrated that the location of residential addresses near intense urban traffic or neighbouring industrial emissions is the main cause of adverse health effects [19]. Feasible solutions for reducing the level of exposure to air pollution are to find alternative routes with lower concentrations of air pollutants while commuting/travelling [20] and to avoid/reduce outdoor activities during “rush hour” and air pollution episodes [21].

Exposure assessment has been defined as “the process of estimating or measuring the magnitude, frequency and duration of exposure to an agent” [22]. The most common method for assessing human exposure to air pollution is to estimate the population-wide annual (long-term) or daily (short-term) average exposure to concentrations using data from fixed monitoring networks and air quality models [23]. Modelling of population exposure is a complex task that is influenced by people’s activity patterns, the characteristics of their residence (e.g., indoor sources, proximity to outdoor sources, and the type of housing), the characteristics of their workplace, their socioeconomic status, and the topography/meteorology in their living and working locations [24]. Every individual has unique activity/patterns that will result in a different exposure to air pollution. In this context, the development of standardized exposure metrics to characterize the multipollutant environment is required to understand the impact of ambient air pollution on human health [25].

Assessing long-term exposure to particulate air pollution and their associated health effects in children is difficult, mainly because of the complex setup required for the personal monitoring of particulate matter and the complex mobility patterns [26,27,28]. New methods for quantifying children’s exposure to various harmful fractions of PM [1,14,29] are explored, as is an assessment of the effects that particle size and associated chemical speciation on health [30,31].

A multi-criteria approach is required to investigate the PM exposure scenarios by integrating spatial and temporal components in environmental epidemiologic investigations [32]. A dedicated geo-information system can improve the spatiotemporal forecasting of pollutant dispersion in urban areas by coupling air pollution mapping and spatiotemporal geostatistical assessments of adverse health effects based on atmospheric numerical models, in situ measurements and epidemiological studies [33,34,35]. Early warning systems have improved the protection of citizens by reducing personal exposure to health risks during critical episodes of heat waves or air pollution [36,37]. Personal monitors have been increasingly used to quantify individual exposure or evaluate the PM traffic loading of various heavy-traffic streets or districts [38,39,40]. Consequently, personal exposure assessment is evolving quickly and the latest advances in Information and Communications Technology have enabled the tracking of individuals while monitoring pollutant concentrations.

In this context, the rationale of the study was to evaluate the effect of PM_2.5_ air pollution on the exacerbation of asthma and respiratory disorders in sensitive children. A multi-criteria approach was used to estimate the potential inhaled doses of PM_2.5_ and the frequency of respiratory symptoms occurring due to PM_2.5_ pollution episodes in Targoviste, a city located in the south of Romania that is impacted by a metallurgical industry and intense road traffic.

It was hypothesized based on the recommendation of paediatric experts that monitoring fever as an indicator of viral infections can facilitate the identification of PM pollution episodes as triggers of respiratory symptoms in sensitive children by excluding from counting the associated health effects when fever occurred. Wheezing and persistent coughing were considered symptoms that may be linked to PM air pollution. Consequently, the duration and frequency of the relevant respiratory symptoms, i.e., persistent coughing, rhinorrhoea, wheezing and fever, were analysed in a selected group of children. The first goal was to establish the potential trigger of symptoms due to PM_2.5_, and the second goal was to evaluate the variability of symptom duration occurring due to outdoor exposure in the analysed group.

The conducted analysis had the following objectives: (1) rank the urban areas based on the monitored concentrations of PM_2.5_; (2) analyse the PM_2.5_ time series establishing the multiannual trends and weekdays/weekend levels of pollution; (3) assess outdoor exposure using air quality maps and GPS tracks of the selected group of 25 sensitive children; (4) estimate the daily outdoor PM_2.5_ doses based on functional probes of children and pollutant concentrations in various locations; and (5) assess the respiratory symptoms in the analysed group.

## 2. Materials and Methods

Quantifying the health impact of PM pollution within an urban area requires an experimental protocol that is based on a logic diagram containing the field survey and laboratory analysis activities, a data processing infrastructure, and correlation with epidemiological information [41].

### 2.1. Site Description

The studies were performed in Targoviste (latitude 44°56′ N, longitude 25°26′ E, altitude 280 m), a Romanian city with approximately 74,000 permanent residents. Historically pollution with PM and heavy metals in the area started in 1973. The industrial emissions have significantly diminished due to the economic constraints starting in 2009. A metallurgical plant for steel production and several metalworking facilities are located to the south, near the city limits. In the city centre, a company produces rigs. A coal thermal plant, which stopped functioning in 2009, as well as some chemical point sources are located 7 km northwest of the city. Some of these industrial facilities have been dismantled, and the buildings were demolished.

Since 2012, when the firm that operated a centralized cogeneration system went bankrupt, domestic heating relies on decentralized systems (mainly small gas boilers and wood stoves). In 2013 and 2014, there was extensive construction work associated with water supply system rehabilitation operations that implied excavations, asphalt stripping, large asphalting operations and rehabilitation of road infrastructure. These operations contributed to the total emissions in the area due to exhaust from equipment and resuspension of dust containing past-emitted heavy metals. Consequently, the main air pollution sources in the area are the intense traffic, decentralized heating during the cold months, and some remaining industrial sources.

### 2.2. Assessment of the Respiratory Symptoms in Children Due to PM_2.5_ in Targoviste, Romania

Recently, people have become increasingly concerned about the adverse health effects from exposure to air pollution via selected routes, especially if they or a member of their family experience respiratory discomforts. The issue is more important in the case of sensitive children that have asthma and asthma-related respiratory disorders. Consequently, our study was developed in line with the latest experimental approaches for quantifying the effects of particulate air pollutants on the inflammatory response and respiratory symptoms in children [42,43,44,45].

The trial was conducted over three months (October–December 2015) and involved the active cooperation of the children’s parents for carefully monitoring the child’s respiratory symptoms and outdoor program. We selected this period because the PM_2.5_ concentrations rise significantly due to domestic heating sources and because the children’s outdoor program is longer than that in January and February, when lower temperatures and frosts occur. One solution for estimating the dose of PM_2.5_ that might be inhaled by the children in a specific area is to use functional probes, the PM levels that occur at various moments of the day, and the time spent outdoors by a specific child at those moments.

We selected the most sensitive children from the database of 111 children who experienced respiratory symptoms elaborated in a previous study [14], while trying to have a reasonable distribution regarding the age, gender, and home address (Table 1).

Other criteria were the will of the parents to cooperate in this study and their knowledge regarding the use of GPS. The 25 selected children who were included in the study presented perturbed respiratory health (i.e., wheezing, asthma, and associated symptoms) and each of them had a file with medical information containing the number of wheezing episodes, number of asthma attacks (with hospitalization), the response to inhalation medication, medication controller, eosinophil count, and serum level of immunoglobulin E (IgE) [46]. The number of selected children was small to maintain optimal control of the experiment and because of the complex logistics involved. The power of the designed experiment computed for a group size of 25 subjects was 0.848 (one tail; effect size = 0.5—medium effect; α = 0.05). A statistical power value of 0.8 is often used in practice [47]. Consequently, the designed experiment showed sufficient statistical power.

Fever was considered a control variable because it is a reliable indicator of viral infections. Consequently, when a fever occurred in a specific child, the corresponding respiratory adverse episode was excluded from counting the air pollution as a potential trigger of respiratory symptoms.

Each parent was asked to complete the prepared sheets containing the following information: date of birth, weight, height, home address, school/kindergarten, medication during the trial, occurrence and duration of respiratory symptoms (i.e., coughing, rhinorrhoea, wheezing, and fever), and daily physical effort. They noted the day when a respiratory event/symptom started and the day when it stopped. Second, they described in a diary the time spent outdoors and the physical activities, thus allowing an estimation of the PM_2.5_ dose at a certain moment. At the end of the study, the GPS tracks were collected and used to aggregate the exposure levels in the analysed group.

### 2.3. Particulate Matter Monitoring

**Figure 1:**
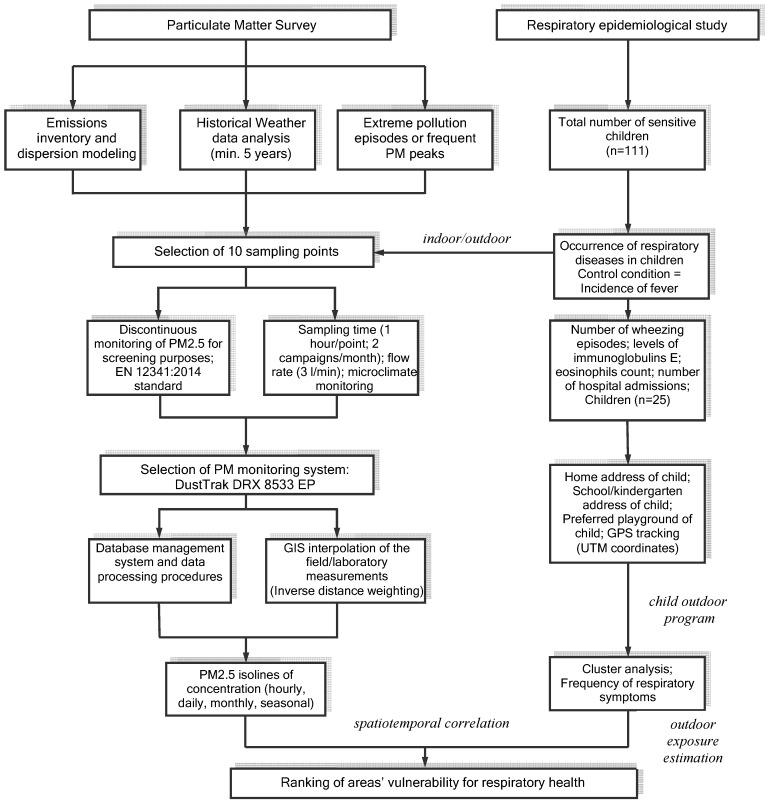
Diagram for quantifying the impacts of particulate matter pollution in urban environments and the respiratory health effects in children used in the current study.

The authors applied a complex procedure for spatiotemporally correlating of PM effects with children’s respiratory health in the Rokidair project [41] (Figure 1). The multi-criteria approach was developed for use with a database consisting of cases of children having respiratory issues, PM_2.5_ records, dispersion modelling results, and meteorological data. This facilitated the production of thematic maps with specific attributes [46].

PM_2.5_ monitoring campaigns were performed twice a month over three consecutive years (2013–2015) at ten representative monitoring points of Targoviste, Romania. The sampling points were established using a top-down approach based on the results of dispersion models, which enabled a quasi-radial spatial dispersal in relation to the town’s shape. Meteorological parameters collected from the Targoviste WMO (153750) station (e.g., air temperature, wind speed and direction, relative humidity and precipitation—hourly average values) were used in the BREEZE^®^ AERMOD dispersion model (Trinity Consultants, Dallas, TX, USA, 2015). Other selection criteria were the proximity of the sampling points to schools, kindergartens and playgrounds and to the monitoring points of the national authorities’ network. DustTrak^TM^ DRX 8533EP with an environmental enclosure (www.tsi.com), which is a precision optical instrument, was chosen for monitoring the PM_2.5_ concentrations at the designated sampling points.

The interpolation of measurements performed at the ten sampling points for screening purposes ensured spatiotemporal covering of the city with modelled PM_2.5_ concentrations. The outcomes of this approach were the personalized air pollution exposure and estimated dose maps. The collected datasets were used to obtain thematic maps using the QGIS software (QGIS Development Team, Open Source Geospatial Foundation Project, 2016, http://www.qgis.org/en/site/), and the data were interpolated using an inverse distance weighting (IDW) algorithm. IDW is widely used to produce isolines of concentrations in air pollution studies [23,42] and is integrated into contouring and surface modelling software packages. Previous findings suggest that the choice of geocoding technique may influence estimated health effects when air pollution exposures are assessed using a fine-scale exposure model [48]. For this study, we used Open Street Maps and ESRI customized layers (e.g., buildings, streets, functional areas) to improve the spatial precision that allowed a cluster analysis of the PM_2.5_ levels on the map of Targoviste.

The urban areas were classified into three categories of pollution level (i.e., 1—highest polluted, 2—middle polluted, and 3—least polluted). There were further coupled with the children’s respiratory illnesses database developed in the Rokidair project with the support of the participating hospitals and family paediatric doctors [14].

In this study, the delimited areas were associated with three groups of children considering the position of their home address in one of the established areas (i.e., 1—A, 2—B, and 3—C).

### 2.4. PM_2.5_ Time Series Analysis

A seasonal analysis of the multiannual time series of PM_2.5_ recorded between 2013 and 2015 was performed to establish the general trend by calculating pair wise the multiannual average concentration and using the resulting average in exponential smoothing (α = 0.1; Mean Absolute Error (MAE) = 4.59).

We then assessed the potential exposure to PM_2.5_ during the day by characterizing each hourly interval. The recorded data were divided into two groups: weekdays and weekends. The daily average of each weekday (Sunday to Monday) was computed using continuous hourly values recorded during a day. Then, the average of subsequent weekday averages was calculated to describe the general pattern of each day of the week over a month. For example, in October 2015, there were four Mondays resulting in four daily averages for computing the average of Monday for October. Later, the monthly average values of each weekday were separated in two groups, Monday–Friday and Saturday–Sunday, to compute the weekday and weekend synthetic averages for each month [21].

Then, the corresponding time series were aggregated, thus facilitating knowledge of the pollutant loads during “working” and “free” days. The rationale for this separation was to obtain a preliminary outlook of the critical hours regarding the exposure of children who attend schools and kindergartens on weekdays and on weekends when they play more in outdoor microenvironments (e.g., preferred playgrounds, courtyards, other urban outdoor facilities).

### 2.5. Outdoor Exposure Assessment

We used the indirect exposure estimation, where the parent carries a smartphone or a GPS recorder, and merged it with the PM_2.5_ concentration maps. The GPS tracks were integrated with air quality maps to estimate the individual exposure along a given track. The advantage of merging the GPS data with an air quality map is that the individual does not need to carry a personal air monitor, thus making the approach applicable for addressing larger groups of people. It is also possible to produce exposure estimates over hypothetical routes or routes that the parents plan to take in the future.

The exposure was computed by averaging the concentration along a line segment and multiplying it by the time spent on this segment, as in Equation (1):

(1) $$X_{ij}=\overset{K}{\underset{k}{\sum }}c_{jk}\times t_{ik}$$

where $X_{ij}$ is the total exposure for person *i* over a specified period of time for pollutant *j*, $c_{jk}$ is the concentration of pollutant *j* in microenvironment *k*, $t_{ik}$ is the residence time of person *i* in microenvironment *k*, and *K* is the total number of microenvironments.

Furthermore, if we know the functional probes of a child, it is possible to estimate the PM_2.5_ dose over a track or during the time spent in a specific microenvironment (Equation (2)):

(2) $$D_{ij}=\overset{K}{\underset{k}{\sum }}r_{ik}\times c_{jk}\times t_{ik}$$

where $D_{ij}$ is the estimated dose, $r_{ik}$ is the respiratory volume (time) in microenvironment *k*, and *K* is the total number of microenvironments. We extracted from the literature the normal values for various ages and biometrics (Table 2).

The standard values regarding the respiratory functions may vary according to the biometric characteristics and the specific effort or activity of a particular child. Sensitive children with respiratory disorders often present lower ventilation rates. The development of specific algorithms to simulate the physiological responses according to the illness of a child in various microenvironments is important for a more accurate assessment. This is important because differences in ventilation will influence the inhaled doses of air pollution. For example, ventilation levels of cyclists are on average two times higher compared to bus and car passengers [50].

### 2.6. Statistical Analysis

SPSS software (SPSS, Chicago, IL, USA, 2011) was used to perform descriptive, associative, and comparative statistics of the data set. The analysis of variance (ANOVA) and multiple range tests (LSD) provided the statistical significance of comparisons. Pearson correlation was used to estimate the strength of the linear relationship between the variables. The odds ratio (OR) [44] was calculated to compare the magnitude of various risk factors for the outcome of interest (e.g., wheezing occurrences in the analysed group).

## 3. Results and Discussion

### 3.1. Assessment of PM_2.5_ Concentrations and Their Spatiotemporal Variability in Targoviste

A reliable assessment of human exposure to environmental agents should incorporate mobility patterns and temporal changes in human behaviours. The temporal dimension is often under-emphasized in exposure assessment studies, due in part to insufficient tools for visualizing and examining temporal datasets [35]. In general, personalized routing relies on traveller’s preferences, which are usually based on different criteria, such as the shortest, fastest, least trafficked, or the least expensive [32]. Including the air pollution criterion in selecting routes could provide better personal protection of health by avoiding the contaminated microenvironments.

**Figure 2:**
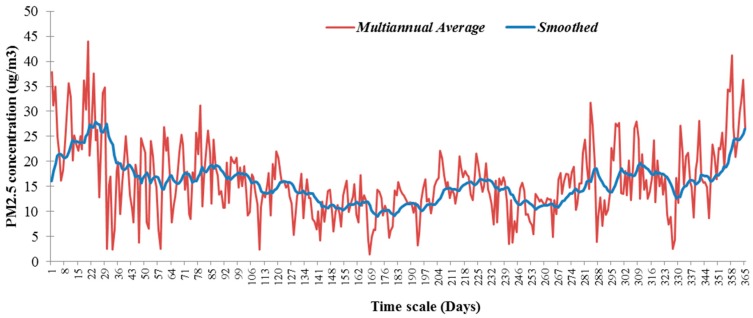
PM_2.5_ multiannual average (16.05 µg·m^−3^) recorded in Targoviste and the trend resulting from exponential smoothing.

Figure 2 shows the result that corresponds to the typical seasonal fluctuation of the PM_2.5_ time series reported in other studies, e.g., [14,27,28]. In this context, the exposure levels are highest in the cold months compared to the warmer ones [51]. However, the time corresponding to the potential outdoor program of a child is longer on warmer days, which means longer exposure to air pollutants and higher inhaled doses. The resulting time series presents sharp increases followed by a rapid decrease throughout the year because the main contribution in the area is the intense traffic. Domestic heating contributes only during cold months. The diminishing of the industrial sources to PM local concentrations is evident by the sawtooth aspect of the time series resulting from night time dispersion.

**Figure 3:**
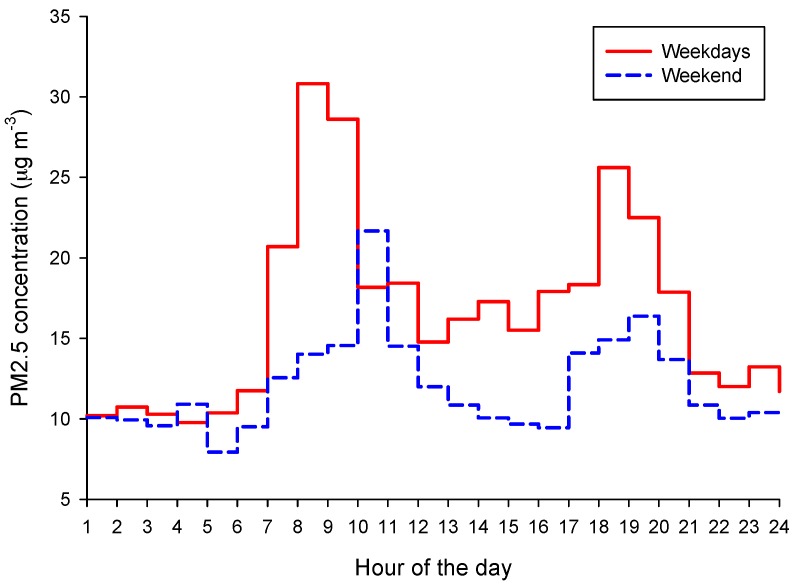
PM_2.5_ trend evaluation during weekdays and the weekend by integrating hourly measurements (average weekdays = 16 µg·m^−3^; average weekend = 12 µg·m^−3^) recorded between October and December 2015, in Targoviste.

Figure 3 shows the evaluation of the PM_2.5_ overall trend for weekdays and weekends by integrating the hourly measurements recorded during the health trial in 2015. The typical evolution of PM_2.5_ on a weekday showed two hourly intervals with elevated levels of concentrations, i.e., between 7 and 10 a.m. and between 6 and 8 p.m. A relatively constant threshold was observed between 10 a.m. and 6 p.m. The PM_2.5_ levels during daytime of the weekend were significantly lower than those on weekdays. The highest peak was observed between 10 and 11 a.m. A significant reduction occurred between 12 a.m. and 5 p.m.

Another increment of PM levels was noticed between 5 and 8 p.m., with a maximum between 7 and 8 p.m. It was found that almost 30% of the daily PM_2.5_ total load occurred between 7 and 11 a.m. during the working weekdays but that 27% was covered in the same interval during the weekend. The PM_2.5_ total load of a weekend day was approximately 73% of an average working day. The presented pattern is related to the main “rush hours” that characterize the commuting mode of the community in Targoviste.

**Figure 4:**
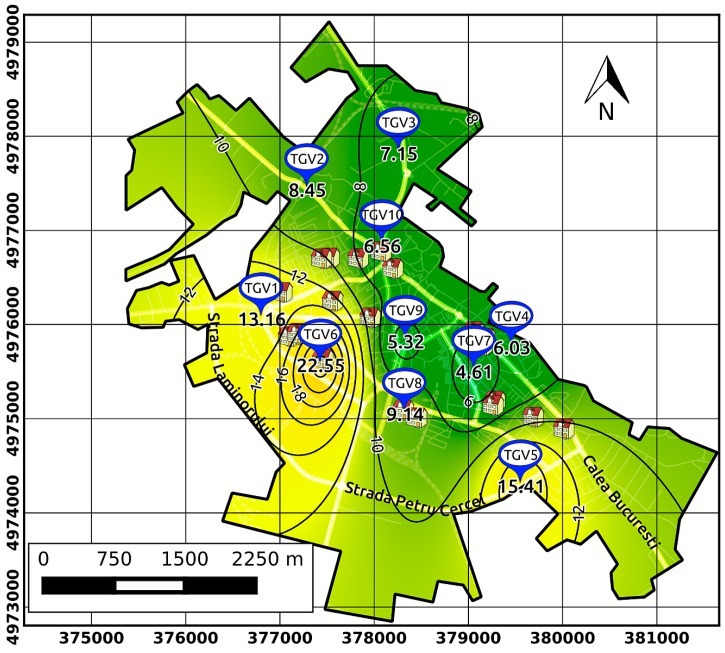
Map of PM_2.5_ concentrations (µg·m^−3^) in Targoviste showing the potential PM_2.5_ levels that resulted from using the inverse distance weighting interpolation (multiannual average 2013–2015); grid with UTM coordinates; TGV 1–10 are sampling points and buildings are schools and kindergartens.

Figure 4 shows the PM_2.5_ interpolation of in situ measurements using the IDW algorithm that provided the isolines of multiannual average concentrations, which allowed for spatial assessment of PM distribution. We observed that the isoline of 10 µg·m^−3^ practically delineates the three areas with different levels of pollution: 1—least polluted ~ 6.5 µg·m^−3^ (centre and north of the city), 2—middle polluted (south-east) ~ 11 µg·m^−3^, and 3—highest polluted ~ 16 µg·m^−3^ (west and south-west).

### 3.2. Assessment of the PM_2.5_ Dose in the Analysed Group of Children

The first step was to correlate the typical program of a child with the hourly recorded PM_2.5_ levels. The second step was to compute the potential exposure by multiplying the time spent outdoors with the corresponding PM concentrations in the area. In the last step, specific physiological characteristics (respiratory parameters and pulse rates) were used to assess the potential inhaled dose of a particular child. The key figures were obtained by interpolating the individual program and physiological characteristics of each child during the study period.

**Figure 5:**
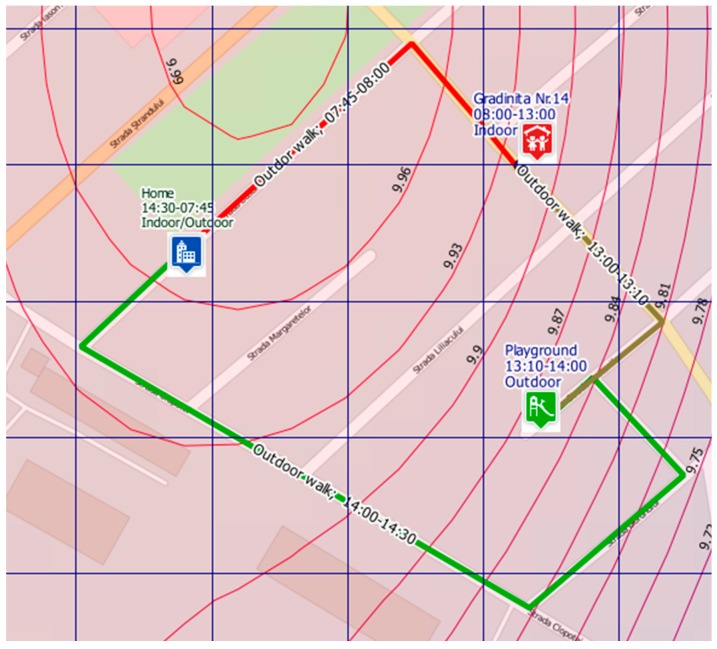
Example of a child’s potential exposure at PM_2.5_ concentrations during a typical weekday (route: home–kindergarten no.14–playground–home) according to the usual program (morning–afternoon); red line—highest exposure; brown line—moderate exposure; green line—lowest exposure; curves represent isolines of PM_2.5_ concentrations for a specific selected hour; simulation performed by Rokidair cyberinfrastructure.

Figure 5 presents an example of the outdoor exposure scenario on a typical weekday according to the usual program of a child (morning–afternoon) using the most common routes (route: home–kindergarten–playground–home).

In our study, we centralized the data acquired from the group of 25 children with respiratory issues, and the results for the potential doses of PM_2.5_ are presented in Table 3. The variations in the ranges of various ages were related to the ventilation rates that were lower than normal values and fluctuated depending on the child’s activity and individual response, the timeframe and location of the outdoor activity, and the commuting mode that significantly shortened the outdoor exposure if car transport was used between locations. There was a significant difference between the estimated doses for the weekdays and the weekend (*p* < 0.05). The estimated ranges were 0.8–14.5 µg·day^−1^ for weekdays and 0.4–6.6 µg·day^−1^ for the weekend. Such amplitudes suggest the complexity that characterizes the evaluation of the PM_2.5_ impact on children with respiratory sensitivity due to the difficulties of correct estimating the real ventilation rate and providing a timely evaluation of personal exposure.

However, the obtained results using air quality maps and statistical indicators of physiological probes provided valuable preliminary information regarding the relationship between outdoor PM_2.5_ levels and the incidence of respiratory symptoms. Some prospective cohort studies in adults found that the range of average ambient PM_2.5_ concentrations is approximately 5–30 μg·m^−3^, resulting in an estimated dose of PM_2.5_ between 90 and 540 µg·day^−1^ from ambient air pollution [52]. Such values include both outdoor and indoor exposures to various sources (e.g., smoking) and are significantly higher than our reported ranges.

The averaged results obtained in the current study for outdoor estimated doses in cold months with reduced outdoor program were related to the children’s weight (0.22–0.26 μg/kg·day) and were in line with previous studies, i.e., 0.2 µg/kg·h [53] and 0.32 μg/kg·day [54]. This shows that urban areas are confronting similar issues concerning PM_2.5_ pollution irrespective of location as long as they are under the impact of conventional traffic (diesel engines) and neighbouring industrial emissions [55].

### 3.3. Assessment of the Respiratory Symptoms Due to Outdoor PM_2.5_ Concentrations

For cases of respiratory diseases in children aged between 1 month and 6 years, the most common symptoms that may be linked to air pollution with PM are wheezing and persistent coughing. Consequently, we analysed the duration of the relevant respiratory symptoms, i.e., persistent coughing (7.48 ± 0.85, 95% CI (6.62–8.33); Coeff. of var. = 49.8%), rhinorrhoea (4.85 ± 0.81, 95% CI (4.03–5.67); Coeff. of var. = 73.2%), wheezing (1.90 ± 0.41, 95% CI (1.49–2.32); Coeff. of var. = 94.9%), and fever (2.10 ± 0.36, 95% CI (1.74–2.47); Coeff. of var. = 75.4%) in the age groups. In practical terms, we can state with 95% confidence that the abovementioned true means are somewhere between the presented intervals. Table 4 shows the recorded averages, standard deviations, and interquartile ranges for each age group.

The overall results showed no significant differences between months regarding the group average for the studied symptoms. However, a decreasing of the frequency and duration of the symptoms occurred with the increasing of age. The 4- to 5-year old children recorded the longest duration of symptoms, except for rhinorrhoea, which suggests that this age interval is the most vulnerable to the exogenous trigger agents (*p* < 0.01) compared to the other groups. This group was the most active in outdoor activities, which might be linked to the increased rate of respiratory symptoms. Furthermore, this is the age for a more accurate diagnostic of asthma in children [3,44].

It is important to mention that all of the children received medication controllers (bronchodilators, anti-allergic and anti-inflammatory drugs) prescribed by a paediatrician. The reported respiratory symptoms occurred despite the use of medication because of exogenous triggers.

Significant correlations of cumulated symptoms (*n* = 75—25 children × 3 months) were observed between the durations of coughing and rhinorrhoea (Pearson *r* = 0.42; *p* = 0.0002; MAE = 2.37), and between rhinorrhoea and fever (*r* = 0.38; *p* = 0.0007; MAE = 2.17).

**Figure 6:**
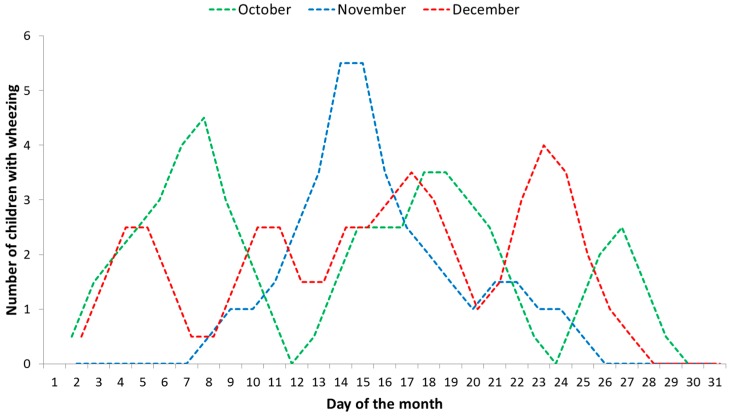
Wheezing occurrences in the analysed group of children (*n* = 25); trend lines that resulted from using the moving average technique: blue line shows the outdoor PM_2.5_ elevated concentrations effect as a potential trigger on wheezing occurrences, whereas the other two are more closely related to a variety of trigger agents.

PM_2.5_ air pollution was found to have a direct positive correlation with wheezing occurrences (Pearson *r* = 0.87; *p* < 0.01) in November 2015. Figure 6 shows the trend lines resulting from using the moving average technique that presented different patterns of wheezing occurrences for each month. October had three distinct periods, November had one, and December did not have a clear separation between periods. We found that in the first week of November 2015, the PM concentrations had risen significantly due to an air mass trajectory from the west (potentially from a thermal power stations’ operation) that was simulated using the HYSPLIT model [56]. The progressive increase of wheezing due to PM_2.5_ is clearly evidenced in the November data set.

Clinically, every child responds in a different way to a variety of trigger agents, to the same trigger agent at various times and even to treatment [3]. Modifications of symptomatology may result from the removal of pollutants or allergens/irritants in areas where children spend time; seasonal changes with variable exposure to pollen or dust; increases in viral infections during the cold months; or lung development and child evolution [16]. In this study, we observed that older children, i.e., those aged 9–10 years, are more resistant to exogenous factors, including PM pollution. Previous studies showed that only when particulate air pollutants have the potential for inducing the pro-inflammatory response, which is dependent on the types and sources of PM, are they able to aggravate the respiratory symptoms of children [43].

In this context, we used the wheezing occurrences in the absence of fever as an indicator of the outdoor PM_2.5_ effect on respiratory symptoms in sensitive children.

**Figure 7:**
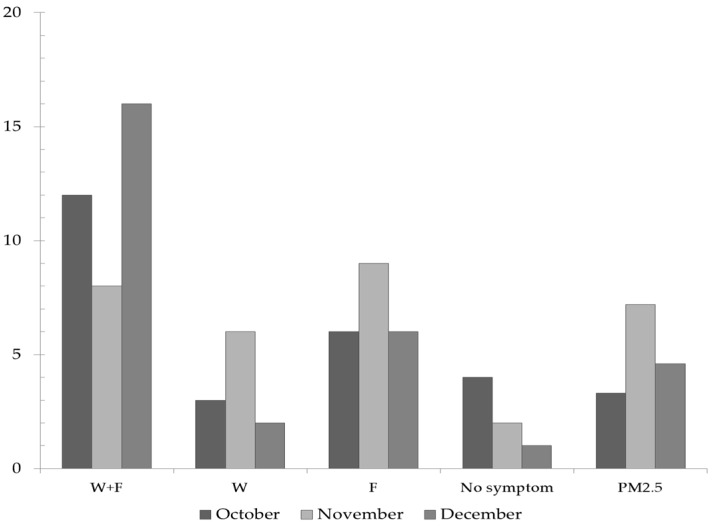
Evolution of wheezing and fever symptoms (number of affected children) in the analysed group (*n* = 25) during October-December 2015 in Targoviste, Romania; W + F = wheezing associated with fever; W—wheezing without fever; F—fever without wheezing; PM_2.5_—frequency of days with diurnal average concentrations >35 µg·m^−3^ (% values were scaled by a factor of 10^–1^).

This indicator allowed a differentiation (OR 0.18, 95% CI (0.04–0.75) *p* = 0.018; exposure associated with lower odds of outcome if PM pollution episodes are not occurring) from the wheezing associated with viral infections. Figure 7 highlights the number of children who presented wheezing in absence of fever in the analysed group. The largest number was recorded in November, the month in which elevated PM_2.5_ concentrations occurred. Furthermore, the children who presented this symptom also spent the longest time outdoors, which also supports this finding. Wheezing associated with fever potentially caused by viral infections mostly occurred in December, a month when only one child did not have symptoms.

Further investigation is needed to assess whether these observations of varying occurrence in symptoms are due to a particular exposure of children in a specific microenvironment. Heart rate monitors using accelerometer data from a mobile application, peak flow meters for peak expiratory flow (PEF) measurements, the use of precise portable monitors (e.g., Casella Microdust Pro) for PM_2.5_ personal monitoring on track, and inclusion of indoor monitoring are required to improve the accuracy of the exposure assessment. This approach can be further refined by including adapted on-line/printed questionnaires that parents can complete for a better characterization of the outdoor program and respiratory failures of a particular child to increase the number of participating subjects.

## 4. Conclusions

The results regarding the development of a multi-criteria approach to assessing the airborne PM_2.5_ effects on respiratory symptoms of sensitive children indicated the importance of incorporating mobility patterns and temporal changes in children’s outdoor programs as well as in the concentrations of contaminants. An environmental mapping system for visualization of the critical areas exposed to air pollution is useful for air quality planning in the region of interest and for protection of sensitive children.

The presented case study provides a versatile solution to assessing the impact of PM_2.5_ on sensitive children’s health. There was a significant difference between the estimated doses for the weekdays and the weekend (*p* < 0.05). The estimated ranges were 0.8–14.5 µg·day^−1^ for weekdays and 0.4–6.6 µg·day^−1^ for the weekend, depending on various factors. A decreasing of the frequency and duration of the symptoms occurred with the increasing of age. The 4- to 5-year old children recorded the longest duration of the symptoms, except for rhinorrhoea, which suggests that this age interval is the most vulnerable to the exogenous trigger agents (*p* < 0.01) compared to the other age groups. PM_2.5_ air pollution was found to have a direct positive correlation with wheezing occurrences (*p* < 0.01) in November 2015.

Monitoring of wheezing occurrences in the absence of fever can be a reliable indicator of the air pollution effect on the exacerbation of asthma and respiratory disorders in sensitive children.

Further development of this approach will provide intelligent support for the children’s health management under the impact of air quality stressors and pressures. Supplemental details are required (e.g., PM_2.5_ chemical speciation, source of emission, other synergic air pollutants) to provide reliable expert advice to the public concerning possible health effects or to help with effective measures for reducing the impact of a pollution episode.

In this context, developing dedicated prevention and research programs is a priority action from the national to international levels. Such programs must track the evolution of these sensitive children in a broader collaboration, including national and international specialized institutions, associations of patients (e.g., European Federation of Allergy and Airways Diseases Patients’ Associations) and, most importantly, the parents. The role of parents must increase because they can allow their children to be interviewed and examined in epidemiological studies.

Parents can provide key information for air quality studies by answering specific questionnaires, observing the child’s status and evolution, and providing information about living conditions and socio-demographic characteristics. They should be integrated as active participants in air pollution—epidemiological studies because they are first to observe the effects of air pollution episodes on child respiratory health.

The developed methodology is intended to serve as a pattern for elucidating the magnitude of PM_2.5_ effects on respiratory symptoms in children in other cities and air-polluted areas. The presented approach requires further epidemiological studies with a larger number of participating children, an improved personal monitoring and fine adjustments to increase its robustness.

## Figures and Tables

**Table 1:** Setup of the experiment to determine the effect of PM_2.5_ air pollution on respiratory symptoms in children.

Indicator	Descriptor
Location	Targoviste, Romania
Study period	Between October 2015 and December 2015 (3 months)
Date of birth	Between January 2005 and December 2013
Age groups	Total of 25 children: Small children: 2–3 years (7); pre-schoolers: 4–5 years (7); school children: 6–8 years (7) and 9–10 (4).
Gender	males (50%); females (50%).
Grouping by home address in a region of the city (sensitive children)	Group A (14)—highest concentrations of PM_2.5_ (~16 µg·m^−3^); Group B (6)—middle concentrations (~11 µg·m^−3^); Group C (5)—lowest concentrations (~6.5 µg·m^−3^)—city centre
Medical records (used to select the children for the trial)	Number of wheezing episodes/asthma attacks and hospitalizations (counted starting with a child’s first consultation/admission in hospital); blood test indicators.
Selection criteria for sensitive children	Number of wheezing episodes; eosinophil count; immunoglobulin E (IgE) serum level; response to inhalation medication.
Control condition	Incidence of Fever
Fields in the observation sheet completed by each parent during the trial	Date of birth; Home address; School/kindergarten; Medication during the trial; height; weight; occurrence of respiratory symptoms i.e., coughing, rhinorrhoea, wheezing, and fever; physical effort
Routes and time spent outdoors	Activities, commuting mode between various locations and timing (diary); GPS tracks using smartphones and Garmin GPS devices

**Table 2:** Functional probes in children for various ages extracted from literature review [3,15,49].

Indicator	Units	1 Week	1 Year	3 Years	5 Years	8 Years	10 Years
Height (average values)	cm	48–52	75	96	100	130	140
Weight (average values)	kg	3	10	14.5	18	26	33
FRC ^1^	mL	75	263	532	660	1174	1546
VC ^2^	mL	100	475	910	1200	1885	2358
Ventilation	mL/min	550	1175	2460	2600	3240	3458
Vt ^3^	mL	17	78	112	130	180	217
Respiratory frequency	breaths/min	30	24	22	20	18	16
Pulse rates ^4^ (low–high)	-	100–160	100–160	90–150	80–140	70–120	60–100
Low-normal systolic blood pressure	-	>60	>70	>75	>75	>80	>90

^1^ Functional Residual Capacity (FRC)—volume of gas that remains in lungs at the end of a normal expiration; ^2^ Vital capacity (VC)—volume of gas that enters in lungs during maximum inhaling; ^3^ Current Volume (Vt)—volume of gas inhaled or exhaled during a ventilation cycle; ^4^ When sleeping, the pulse rates of a child may be 10% lower.

**Table 3:** Outdoor doses of PM_2.5_ estimated in the analysed group of children (µg·day^−1^) between October and December 2015 in Targoviste, Romania; Rng—range, SD—standard deviation, IQR—interquartile range.

Age (Years)	2–3			4–5			6–8			9–10		
	*Rng*	*SD*	*IQR*	*Rng*	*SD*	*IQR*	*Rng*	*SD*	*IQR*	*Rng*	*SD*	*IQR*
Weekdays												
Route to kindergarten/school	0.85–1.85	0.7	0.5	0.90–1.95	0.7	0.5	1.12–2.43	0.9	0.7	1.19–2.59	1.0	0.7
Play after school	1.09–2.36	0.9	0.6	1.15–2.50	1.0	0.7	1.43–3.11	1.2	0.8	1.53–3.32	2.0	0.9
Play in the afternoon	1.36–2.95	1.1	0.8	1.44–5.72	3.0	2.1	1.79–7.78	4.2	3.0	1.91–8.65	4.8	3.4
Estimated dose (day)	3.29–7.16	2.7	1.9	3.48–10.2	4.7	3.3	4.34–13.32	6.3	4.5	4.63–14.56	7.0	5.0
Weekend												
Morning walk	0.41–2.51	1.5	1.1	0.43–2.65	1.6	1.1	0.54–3.30	2.0	1.4	0.57–3.53	2.1	1.5
Play in the afternoon	0.68–1.48	0.6	0.4	1.01–1.56	0.4	0.3	1.61–2.92	0.9	0.7	1.72–3.11	1.0	0.7
Estimated dose (day)	1.09–3.99	2.0	1.4	1.44–4.21	1.9	1.3	2.15–6.22	2.8	2.0	2.29–6.64	3.1	2.1

**Table 4:** Centralized results of the respiratory symptoms duration (days); respiratory symptoms are typed with bold text—averages recorded in the analysed group of children (*n* = 25) between October and December 2015 in Targoviste, Romania; SD—standard deviation; IQR—interquartile range.

Age (Years)	2–3 (*n* = 7)	4–5 (*n* = 7)	6–8 (*n* = 7)	9–10 (*n* = 4)	Group Average	*SD*
**Persistent Coughing**						
October	8.7	9.1	6.0	5.6	7.4	*1.8*
*SD*	*4.6*	*3.7*	*1.8*	*3.5*	-	*-*
*IQR*	*4.5*	*4.0*	*2.5*	*3.7*	-	*-*
November	9.3	9.0	6.9	4.7	7.5	*2.1*
*SD*	*3.9*	*5.5*	*3.2*	*2.3*	-	*-*
*IQR*	*6.0*	*2.0*	*1.5*	*2.9*	-	*-*
December	7.3	7.9	6.9	5.8	6.9	*0.9*
*SD*	*5.4*	*2.7*	*1.8*	*3.0*	-	-
*IQR*	*2.0*	*3.5*	*2.0*	*2.1*	-	-
*Total average*	**8.4**	**8.7**	**6.6**	**5.4**	**7.3**	*-*
**Rhinorrhoea**						
October	4.6	5.7	5.4	3.5	4.8	*1.0*
*SD*	*3.0*	*4.6*	*2.8*	*2.2*	-	*-*
*IQR*	*3.5*	*5.5*	*3.0*	*2.0*	-	*-*
November	5.6	4.3	4.7	2.1	4.2	*1.5*
*SD*	*3.7*	*2.0*	*4.5*	*1.6*	-	*-*
*IQR*	*1.5*	*2.0*	*1.5*	*3.2*	-	*-*
December	6.3	5.0	5.6	3.1	5.0	*1.4*
*SD*	*5.4*	*3.3*	*4.5*	*1.6*	-	*-*
*IQR*	*3.0*	*2.0*	*3.0*	*2.0*	-	-
*Total average*	**5.5**	**5.0**	**5.2**	**2.9**	**4.7**	*-*
**Wheezing**						
October	2.7	1.4	2.4	1.2	1.9	*0.7*
*SD*	*1.6*	*1.9*	*2.4*	*1.0*	-	*-*
*IQR*	*1.5*	*3.0*	*4.5*	*1.6*	-	*-*
November	1.7	2.3	1.4	0.7	1.5	*0.7*
*SD*	*2.9*	*1.3*	*1.8*	*0.6*	-	*-*
*IQR*	*2.0*	*1.0*	*3.0*	*0.9*	-	*-*
December	1.6	3.1	2.6	0.7	2.0	*1.1*
*SD*	*1.5*	*0.7*	*2.0*	*0.6*	-	-
*IQR*	*3.0*	*0.5*	*3.0*	*0.9*	-	-
*Total average*	**2.0**	**2.3**	**2.1**	**0.9**	**1.8**	*-*
**Fever**						
October	2.1	2.9	1.6	1.1	1.9	*0.8*
*SD*	*1.6*	*1.7*	*1.5*	*1.0*	-	*-*
*IQR*	*2.0*	*2.0*	*2.0*	*1.6*	-	*-*
November	3.3	1.9	1.4	0.5	1.8	*1.2*
*SD*	*1.7*	*1.9*	*1.4*	*0.2*	-	*-*
*IQR*	*2.5*	*3.5*	*2.5*	*0.2*	-	*-*
December	2.0	3.3	2.1	1.9	2.3	*0.7*
*SD*	*1.2*	*1.6*	*1.6*	*1.0*	-	-
*IQR*	*1.5*	*2.0*	*2.0*	*1.6*	-	-
*Total average*	**2.5**	**2.7**	**1.7**	**1.2**	**2.0**	-
